# Solution Processed Zn_1−*x*−*y*_Sm*_x_*Cu*_y_*O Nanorod Arrays for Dye Sensitized Solar Cells

**DOI:** 10.3390/nano11071710

**Published:** 2021-06-29

**Authors:** Muhammad Saleem, Ali Algahtani, Saif Ur Rehman, Muhammad Sufyan Javed, Kashif Irshad, Hafiz Muhammad Ali, Muhammad Zeeshan Malik, Amjad Ali, Vineet Tirth, Saiful Islam

**Affiliations:** 1 Institute of Physics, The Islamia University of Bahawalpur, Bahawalpur 63100, Pakistan; 2 Mechanical Engineering Department, College of Engineering, King Khalid University, Abha 61411, Asir, Saudi Arabia; alialgahtani@kku.edu.sa (A.A.); vtirth@kku.edu.sa (V.T.); 3 Research Center for Advanced Materials Science (RCAMS), King Khalid University, Guraiger, Abha 61413, Asir, Saudi Arabia; 4 Department of Physics, COMSATS University Islamabad Lahore Campus, Lahore 54000, Pakistan; saifrehman@cuilahore.edu.pk; 5 School of Physical Science and Technology, Lanzhou University, Lanzhou 730000, China; 6 Interdisciplinary Research Center in Renewable Energy and Power Systems (IRC-REPS), King Fahd University of Petroleum & Minerals, Dhahran 31261, Eastern Province, Saudi Arabia; kashif.irshad@kfupm.edu.sa (K.I.); hafiz.ali@kfupm.edu.sa (H.M.A.); amjad.ali@kfupm.edu.sa (A.A.); 7 Mechanical Engineering Department, King Fahd University of Petroleum and Minerals, Dhahran 31261, Eastern Province, Saudi Arabia; 8 School of Electronics and Information Engineering, Taizhou University, Taizhou 318000, China; 202106g@tzc.edu.cn; 9 Civil Engineering Department, College of Engineering, King Khalid University, Abha 61413, Asir, Saudi Arabia; sfakrul@kku.edu.sa

**Keywords:** optical properties, Zn_1−*x*−*y*_Sm*_x_*Cu*_y_*O nanorod arrays, dye-sensitized solar cells, I-V measurements

## Abstract

Cu- and Sm-doped ZnO nanorod arrays were grown with 1 wt% of Sm and different weight percents (0.0, 0.5, 1.0 and 1.5 wt%) of Cu by two-step hydrothermal method. The influence of Cu concentration and precursor of Sm on the structural, optical and photovoltaic properties of ZnO nanorod arrays was investigated. An X-ray diffraction study showed that the nanorod arrays grown along the (002) plane, i.e., c-axis, had hexagonal wurtzite crystal structure. The lattice strain is present in all samples and shows an increasing trend with Cu/Sm concentration. Field emission scanning electron microscopy was used to investigate the morphology and the nanorod arrays grown vertically on the FTO substrates. The diameter of nanorod arrays ranged from 68 nm to 137 nm and was found highly dependent on Cu concentration and Sm precursor while the density of nanorod arrays almost remains the same. The grown nanorod arrays served as photoelectrodes for fabricating dye-sensitized solar cells (DSSCs). The overall light to electricity conversion efficiency ranged from 1.74% (sample S_1_, doped with 1 wt% of Sm and 0.0 wt% of Cu) to more than 4.14% (sample S_4_, doped with 1 wt% of Sm and 1.5 wt% of Cu), which is 60% higher than former sample S1. The increment in DSSCs efficiency is attributed either because of the doping of Sm^3+^ ions which increase the absorption region of light spectrum by up/down conversion or the doping of Cu ions which decrease the recombination and backward transfer of photo-generated electrons and increase the electron transport mobility. This work indicates that the coupled use of Cu and Sm in ZnO nanorod array films have the potential to enhance the performance of dye-sensitized solar cells.

## 1. Introduction

One-dimensional (1D) nanostructures have been an emerging class of oxide materials for the last few years because of their outstanding electrical and optoelectronic properties. These excellent properties of 1D nanostructures make them suitable for potential applications in piezoelectric, gas sensing and solar cell devices [1,2,3,4]. Currently, there has been a huge interest in 1D ZnO nanostructures (nanowire/nanotube/nanorod arrays) as photoanodes in dye-sensitized solar cells because of low toxicity, easily reproducibility, facile and low temperature synthesis methods [5]. Furthermore, 1D geometry of ZnO provides direct pathways, necessitating for faster transport of photo-generated electrons from the point of injection to the surface of the collecting electrode [6,7]. In other words, in 1D ZnO architectures, the mobility of charge carriers is higher, recombination possibility is lower, and the charge carriers would not suffer any grain boundary scattering [8]. However, the widespread use of 1D ZnO in DSSCs is still limited because of reduced surface area which presented comparatively low conversion efficiency when compared to the standard TiO_2_ nanoparticle film based DSSC. Basically, DSSCs consists of four key components, conductive substrate + nanostructured semiconductor (working electrode), counter electrode, redox-mediator (electrolyte) and visible-light absorber dye [9]. Different research groups are working on different parts of the DSSCs. Some groups are performing their research on working electrodes (coating of different materials like TiO_2_, SnO_2_, Nb_2_O_5_ ZnO and so on, with different morphologies (e.g., 1D, 2D, 3D) on various substrates) [10,11,12], as well as on counter electrodes (such as Pt/C coated, CoS, Au/GNP, alloys like FeSe and CoNi_0.25_) [13,14]. Other research groups are working on dyes (organic, inorganic and natural dyes) [15,16], as well as on electrolytes (liquid, quasi-solid (gel/paste-like/membranes), solid and water-based electrolytes) [17,18]. From the aforementioned components, working electrodes play a crucial role in the performance of DSSCs. Among these semiconductor materials, the overall light conversion efficiency of 1D ZnO nanowire/nanotube/nanorod arrays based DSSCs remains around 4.7% [19]. There are several reasons behind this limited efficiency. Firstly, 1D ZnO photoanodes inherently have low light harvesting capability. Although, after loading N719/N749 dye (band gap 1.8 eV) the capability of photoanodes increased and can function under the visible-light portion. It is well-known that the largest part of the light spectrum consists of ultraviolet (UV) and near-infrared (NIR) light [20]. Unfortunately, DSSCs cannot absorb 50% of solar irradiation in the UV and NIR regions. Secondly, 1D nanostructures have a high aspect ratio, but the dye loading capacity is significantly lower because of the small surface area between nanowire/nanotube/nanorod arrays. Thirdly, low dye adsorption in 1D geometry is either because of the electrostatic repulsion of dye molecules at the surface of the semiconductor or the backward transfer of electrons. These drawbacks decrease the short circuit current and open circuit voltage in DSSCs and contribute significantly to the bottlenecking of the performance of the device. To address these lapses, many researchers tried to modify the ZnO nanostructures using different strategies including doping with 3d transition metals such as Mn, Co, Cr, Fe, Fr, Cu [21,22,23,24], or intra 4f transition rare earth (RE) metals such as La, Nd, Sm Ce, Er, Yb, Dy [25,26], as well as the coupled use of these metals. Recently, doping with RE gets paramount importance for the researchers because of their optical characteristics in the intra 4f transition [26]. Doping of zinc oxide lattice with rare earth ions resolves the problems related to non-absorbable light spectrum through up/down conversion of NIR and UV radiation to visible wavelength region [27,28]. Doping of ZnO nanorod arrays with Cu ions may decrease the recombination of photo-generated electrons and form a blocking layer to stop the backward transfer of electron. Moreover, Cu forms new energy levels between the valence and conduction band to increase the electron mobility which in turn increase the performance of DSSCs. The doping of ZnO with coupled metals modifies the flat-band potential auspiciously to enable more efficient injection of charge carriers at the interface of ZnO and dye [29,30,31,32,33]. To the best of our knowledge, ZnO has been modified independently with rare earth or transition metal ions, although the coupled use of Cu and Sm has not been reported yet.

In this context, ZnO is used as host material, Cu and Sm are the doping materials. We have discussed the optical, structural and photovoltaic properties of Cu-and Sm-doped ZnO nanorod arrays first time to boost the efficiency of dye-sensitized solar cells. In this regard, a plethora of publications have been published [31,32,33], showing doping with Au/Sm/Eu/Ce of ZnO/TiO_2_ separately. Although, none of these reports explain the combined effect of Cu and Sm doping in ZnO nanorod arrays based DSSCs. The effect of Sm and Cu doping on ZnO nanorod arrays was studied with a fixed concentration of Sm (1 wt%) and different concentrations of Cu (0.0, 0.5, 1.0 and 1.5 wt%). Effects of coupled use of Cu and Sm on the material characteristics of ZnO nanorod arrays and the performance of dye-sensitized solar cells were investigated in detail. The results show that the DSSC fabricated with 1 wt% of Sm and 1.5 wt% of Cu doped ZnO nanorod arrays (sample S_4_) exhibit better efficiency compared to that of DSSC fabricated with 1 wt% of Sm and 0.0 wt% of Cu doped ZnO nanorod arrays (sample S_1_). This result indicates that the Cu-and Sm-doped ZnO nanorod arrays have promising applications in the field of dye-sensitized solar cells, and the ZnO nanorod arrays synthesized with coupled use of RE Sm and transition metal Cu are efficient for the improvement of DSSC’s performance.

## 2. Experimental Details

### 2.1. Chemicals

Analytical grade zinc acetate dehydrate $\left(\mathrm{Zn}\;{\left({\mathrm{CH}}_{3}\mathrm{COOH}\right)}_{2}\cdot 2H_{2}O\right)$ ZnAc), cupric acetate $\left(\mathrm{Cu}\;{\left({\mathrm{CH}}_{3}\mathrm{COO}\right)}_{2}\cdot H_{2}O\right)$ (CuAc), samarium acetate trihydrate $\left(\mathrm{Sm}\;{\left(C_{2}H_{3}O_{2}\right)}_{3}\cdot 3H_{2}O\right)$ (SmAc) hexamethylenetetramine $\left(C_{6}H_{12}N_{4}\right)$ (HMTA), ammoniumhydroxide *(*NH_4_OH), polyethyleneimine ${\left(C_{2}H_{5}N\right)}_{n}$ (PEI) and ethanol were purchased from Sigma Aldrich, Lahore, Pakistan and used without any prior treatment.

### 2.2. Methods

The growth of Cu- and Sm-doped ZnO nanorod arrays was completed in two steps: (1) spin coating of seed layer and (2) hydrothermal method used for the growth of nanorod arrays.

#### 2.2.1. Spin Coating of Seed Layer

To prepare the seed solution, 0.005 M of zinc acetate powder was taken, it was dissolved in 50 mL of ethanol by magnetic stirring. For uniform and full mixing, the solution was continuously stirred at room temperature for 1 h. Before the ZnO seed layer deposition, fluorine-doped tin oxide conducting glass (FTO, sheet resistance 8 Ω/cm^2^) substrates were cleaned ultrasonically with acetone, isopropyl alcohol and ethanol for 10 min each, respectively. The pretreatment of FTO substrates ware taken at room temperature and dried in hot air with hair dryer. The ethanolic precursor seed solution was spin coated on conducting side of fluorine-doped tin oxide (FTO) substrates at 3000 rpm for 30 s. After each layer deposition, the substrates were heated in an electric oven at 100 °C for 10 min. The heated substrates were removed from the electric oven and cooled down naturally at room temperature before coating the next layer. This process was repeated three times to get ~120 nm thick seed layer. Finally, the ZnO spin-coated seed layer films were annealed at 400 °C for 30 min to convert zinc acetate to ZnO nanocrystals. These seed layers act as uniform nucleation sites for the growth of nanorod arrays.

#### 2.2.2. Nanorod Arrays Growth

**Figure 1:**
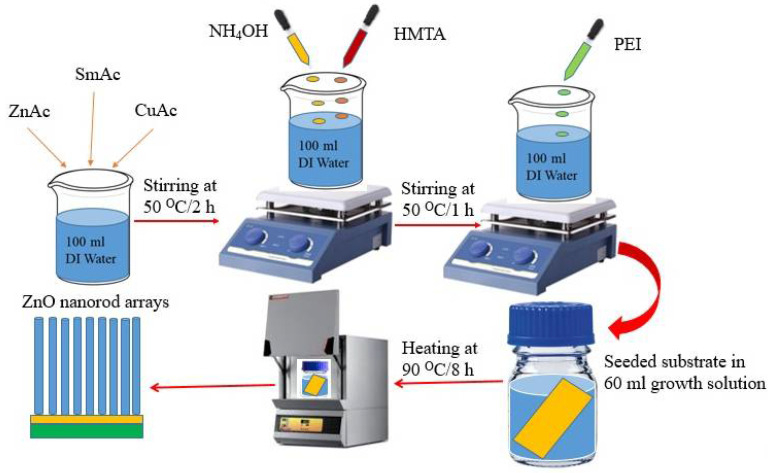
Schematic preparation of Cu- and Sm-doped ZnO nanorod arrays.

The detailed synthesis procedure of Cu- and Sm-doped ZnO nanorod arrays shown in Figure 1 was as follows: 100 wt% of zinc acetate, 1 wt% of samarium acetate and (0.0, 0.5, 1.0 and 1.5 wt%) of cupric acetate, as given in Table 1. These materials were used as starting materials and dissolved in deionized water in a beaker with constant stirring for 2 h at 50 °C. To this solution, 15 mL of hexamethylenetetramine (HMTA) ware dropped under magnetic stirring as a stabilizer. Then, 10 mL of ammonium hydroxide (NH_4_OH) solution was added slowly in the aqueous solution to acquire pH in the range of 7 to 8. Finally, 5 mL of PEI solution was added to the prepared solution and continued stirring at 50 °C for another 1 h. After this step, 60 mL of obtained solutions were transferred to 100 mL glass bottles. The same procedure was repeated to prepare the solution of other concentrations (0.5, 1.0 and 1.5 wt%). Although, the concentration of zinc acetate was regulated according to the concentration of dopant (Cu). For more detail, see Table 1 to clarify the concentration and precursor for each sample (S_1_ to S_4_). Typically, the seeded substrates were placed tilted in glass bottles with solutions in four groups with different concentrations of Cu (0.0, 0.5, 1.0 and 1.5 wt%) and 1 wt% concentration of Sm. These glass bottles were placed in a muffle furnace at 90 °C for 8 h and then allowed to cool at room temperature naturally. In the last step, ZnO nanorod arrays grown FTO substrates were removed from the glass bottles and washed with ultrapure water and annealed in air for 1 h at 300 °C.

### 2.3. Dye-Sensitized Solar Cell Fabrication

After annealing and cooling to 100 °C, the warm Cu- and Sm-doped ZnO nanorod arrays films of average area 0.25 cm^2^ were sensitized into a 0.5 mM solution of dye N719 in acetonitrile and retained in dark overnight for the dye adsorption process. When the dye sensitization is completed, the photoelectrodes were taken out from the dye solution and washed with acetonitrile in order to remove extra dye. Afterwards, the dye loaded photoelectrodes were dried in air for 30 min. Platinum (Pt)-coated FTO glasses of the similar area were used as counter electrodes. Each dye loaded photoanode and the counter electrode were then sealed with the help of 30 μm thick Surlyn frame. The assembled cells were then filled with iodide/triiodide $I^{-}/I_{3}^{-}$ electrolyte, which consists of 0.5 M lithium iodide (LiI), 0.05 M iodine (I_2_) and 0.5 M 4-tertbutylpyridine in acetonitrile. The liquid electrolyte was injected with a dropper through one of two small holes on the counter electrode, which are drilled with the help of a table drill machine. Finally, the two holes were wrapped by a thin glass cover slide in order to prevent the leakage of electrolyte out of the cell. Four devices (DSSCs) were fabricated for each sample and characterized. Only slight errors were observed in the cell parameters and these errors were listed in Table 3 by means of the standard deviation.

### 2.4. Characterization and Measurements

The structural characterization of the as grown nanorod arrays was performed using X-ray diffraction Bruker D8 (Bruker AXS, WI, USA) with Cu Kα radiations (*λ* = 0.154178 nm) at a scanning rate of 0.02°/s from 20 to 80 degrees. The morphology of hydrothermally grown nanorod arrays was investigated by using a scanning electron microscope (SEM, JEOL, JSM-6301F, Chicago, IL, USA). After dye-loading, optical absorption of the photoelectrodes was recorded by UV-Vis spectrophotometer from Ocean optics (Micropack DH-2000, Birlen, Germany). DSSCs J-V characteristic were measured by a Keithley 2450 source meter(SMU 2450, Tektronix, Beaverton, OR, USA) under 1 sun illumination AM 1.5 G (air mass 1.5 global 100 mW/cm^2^). To check the stability of DSSCs, the cells were saved for two months in the dark at room temperature. The stability test was performed every week by measuring the I-V curves.

## 3. Results and Discussion

### 3.1. Crystal Structure of Nanorod Arrays

**Figure 2:**
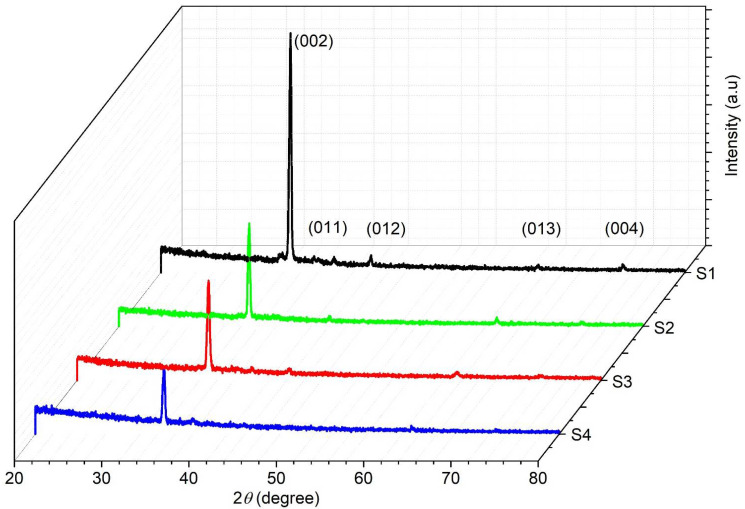
XRD spectra of Cu- and Sm-doped ZnO nanorod arrays.

**Figure 3:**
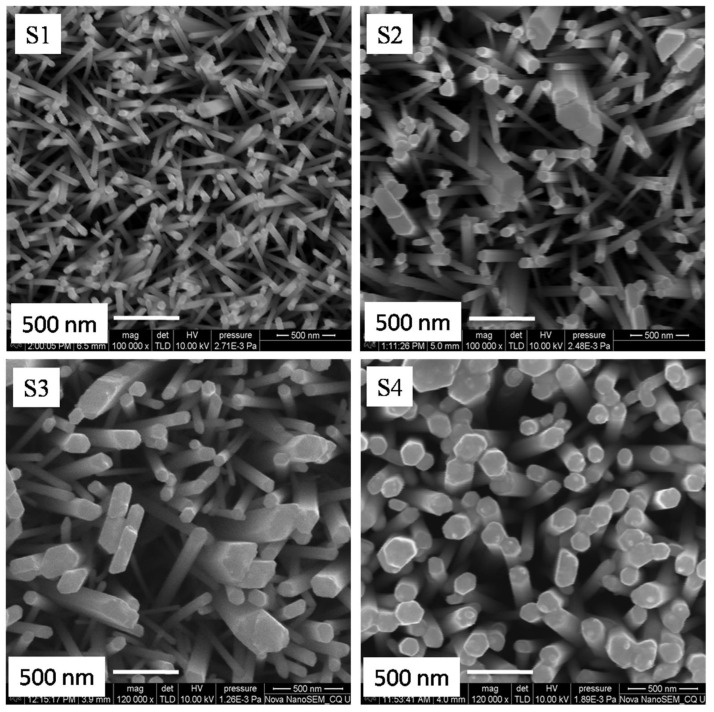
SEM images of Cu- and Sm-doped ZnO nanorod arrays.

Figure 2 illustrates the XRD patterns of Cu- and Sm-doped ZnO nanorod array films. The very strong diffraction peak corresponding to (002) plane of ZnO at 2*θ* = 34.35° is observed in all samples. This peak confirms the hexagonal wurtzite phase of ZnO nanorod arrays in all samples and reveals the fastest growth along the c-axis due to the lowest surface free energy. The grown nanorod arrays are vertically well-aligned to the substrate surface as it is clear from SEM images in Figure 3. In addition, four low intensity peaks corresponding to ZnO (011), (012), (013) and (004) planes are also observed at 2*θ* = $36.65^{{}^{\circ}},\;47.14^{{}^{\circ}},$ 63.31° and 73.32°, respectively (JCPDS card no. 004-3700). No diffraction peaks of Cu/Sm oxides/sub-oxide are traced in the XRD patterns of ZnO nanorod arrays because of small concentration of dopants. This result depicts the successful substitution of Cu^2+^/Sm^3+^ ions into the Zn^2+^ sites without affecting the crystal structure of ZnO. It is clearly noticed that there is a slight shift in peak position towards higher angle in all samples. The shifting in peak position might be due to the shrinkage in ZnO crystal lattice by the incorporation of Cu^2+^/Sm^3+^ ions [27,34]. This shifting can also be attributed to the difference in ionic radii of Zn^2+^ (0.74 Å), Cu^2+^ (0.73 Å) and Sm^3+^ (0.96 Å). Due to the difference in ionic radii, it is expected that the length of the c-axis will be shorter when Cu/Sm atoms are replaced into Zn sites in the crystal lattice [35,36]. In order to explore the influence of Cu concentration and precursor of Sm on the crystallinity of ZnO nanorod arrays, the intensity of (002) peak was observed. For this purpose, crystal size (*D*), compressive strain (*ε*), d-spacing (*d*) and dislocation density (*δ*) were calculated for ZnO (002) peak using Equations (1)–(4) and tabulated in Table 2 [37]:

(1) $$Crystallite\;size\;\left(D\right)=\frac{k\lambda }{\beta cos\;\theta }$$

(2) $$Compressive\;strain\;\left(\varepsilon \right)=\frac{\beta cos\;\theta }{4}$$

(3) $$d-spacing\;\left(d\right)=\frac{n\lambda }{2sin\;\theta }$$

(4) $$Dislocation\;density\;\left(\delta \right)=\frac{1}{D^{2}}$$

where *D* is crystallite size, *k* is constant (its value is 0.9), *λ* wavelength of X-ray (typically 1.5418 Å for Cu Kα) and *β* represents full width at half maximum (FWHM). As reported in the previous literature, the nominal content of Cu/Sm has a significant effect on the ZnO crystal [19,23,24,25,26]. In this work, the precursor of Sm is too small to alter the morphology as is clear from the XRD of sample S_1_. It means that the broadening in width and weakening in the intensity of the peak is due to the substitution of Cu/Sm ions. As the doping concentration of Cu increases from 0.5 wt% to 1.5 wt%, the intensity of (002) peak drops gradually regardless of the Sm precursor as shown in Figure 2. The decrease in (002) peak is more pronounced than the other four (011), (012), (013) and (004) peaks. When the intensity of (002) peak decreases, FWHM increases, as the resulting grain size decreases. This indicates that the drop in (002) diffraction peak is due to the replacement of Cu^2+^/Sm^3+^ ions in Zn^2+^ ions, which restrains the crystal growth of ZnO [38]. Therefore, doping of Cu^2+^/Sm^3+^ may act as an inhibitor for the growth of ZnO along the (002) plane [39]. The same inhibitory trend in crystal growth was also enumerated in other transition and rare earth ions doped ZnO thin films. This decrease in peak position strongly depends on the presence of the lattice distortion, strain and defects induced by the slight substitution of Cu^2+^/Sm^3+^ [40]. The compressive strain is produced during the substitution of Cu/Sm impurities into ZnO lattice and increases with dopant concentration. The decrease in crystallite size and improvement in strain presents defects in the ZnO lattice. It is evident that there is more compressive strain in the samples (S_2_, S_3_ and S_4_) at higher doping level [38]. Dislocation density (*δ*) is a measure of the number of defects appeared in Cu^2+^/ Sm^3+^ ions doped ZnO. Dislocation density defines length of dislocated lines per unit volume of the crystal and calculated using Equation (4). Dislocation density increases with the increase in Cu concentration and Sm precursor. The substitution of Cu^2+^ (0.73 Å) and Sm^3+^ (0.96 Å) with Zn^2+^ ions (0.74 Å) increased defects in the ZnO host lattice. The crystal defects produced in ZnO by doping with Cu/Sm can be calculated from microstrain (*ε*) and dislocation density (*δ*) that increased with the increase in Cu concentration and precursor of Sm. The substitution of Cu/Sm impurities produces cationic vacancies in the ZnO host lattice, and these cationic vacancies decreased the average crystallite size and increased dislocation density, as is clear from Table 2.

### 3.2. Surface Morphology of Nanorod Arrays

**Figure 4:**
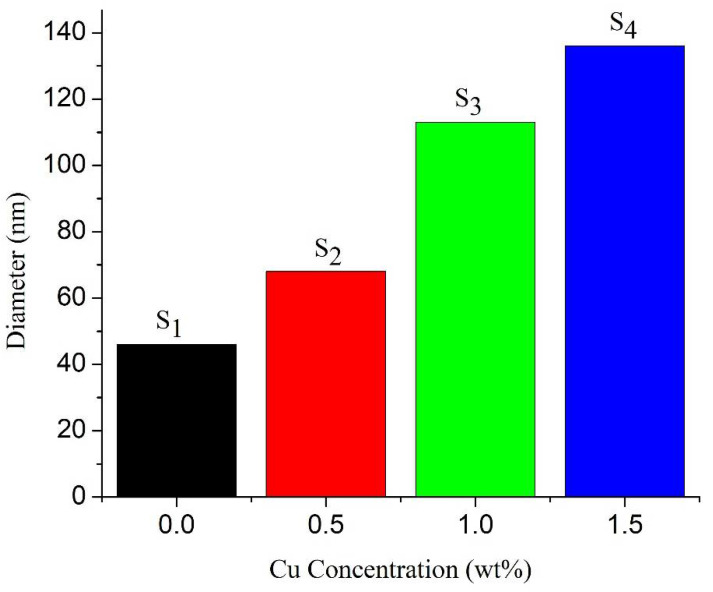
Diameter vs. Cu concentration of ZnO nanorod arrays.

Figure 3 depicts the surface morphologies of Cu- and Sm-doped ZnO nanorod array films. SEM micrograph Figure 3 (Samples S_1_ to S_4_) shows nanorod arrays with different diameters and density because the diameter and density of nanorod arrays depends on Cu concentration and Sm precursor. One can see that when the concentration of Cu was 0.0 wt% and Sm 1 wt%, the as-grown nanorod arrays (sample S_1_) were randomly oriented with varying rod sizes of mean diameter 46 nm. However, when the precursor of Sm was 1 wt% and the concentration of Cu increased from 0.5 wt% to 1.0 wt%, nanorod arrays and grader type morphology (samples S_2_ and S_3_) obtained with collapsing head and different rod sizes. The mean diameter of samples S_2_ and S_3_ was 68 nm and 113 nm, respectively, and the morphology shows little rough surface. When the concentration of Cu increased to 1.5 wt%, dense and well-defined nanorod arrays grow perpendicular to the surface of the substrate with uniform morphology and size and an average diameter of 136 nm. The density of nanorod arrays almost remains the same, leading to a large surface area for more dye anchoring and light harvesting. Tyona and Dom et al. [34,41] have explained that the escalation in Cu/Sm content not only increases the carrier concentration, but also increases the mobility in the conduction band of ZnO. The increment in carrier concentration and mobility reduces the crystallographic defects and increases the crystalline quality of the ZnO film, as can be seen from sample S_4_. This novel morphology is appropriate for DSSC applications. Furthermore, pH value and the nucleation sites of growth solution have a great impact on the diameter and density of nanorod arrays. Babikier et al. [38] proposed that, during the growth of ZnO nanorod arrays, Cu/Sm impurities can increase the density of nucleation sites that boost the growth rate. As the growth rate increases, coalescence between the nanorods takes place, which leads to the formation of longer nanorod arrays with uniform diameter (sample S_4_), as shown in Figure 4. On further increasing the dopants’ concentration, nanorod arrays start overlapping with each other and the morphology obtained with less surface area which decreases the device performance. The photoelectrons take more time to reach the substrate surface because of the hopping mechanism and the cell shows lower efficiency.

### 3.3. Optical Measurement of Nanorod Arrays

**Figure 5:**
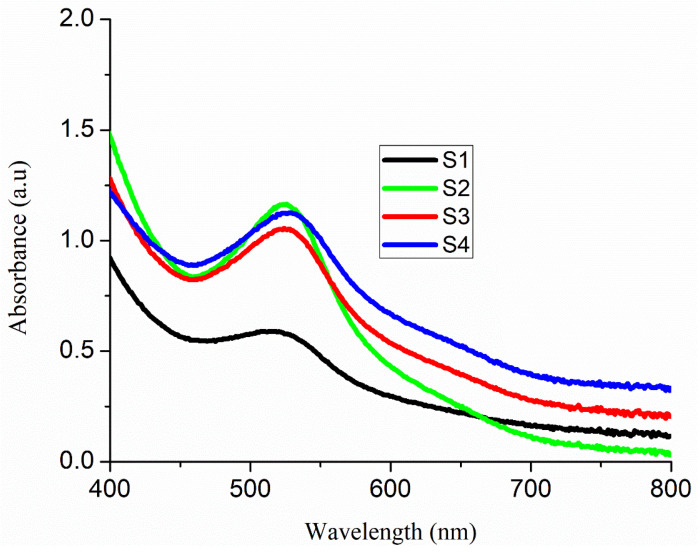
Absorbance spectra of Cu- and Sm-doped ZnO nanorod arrays.

**Figure 6:**
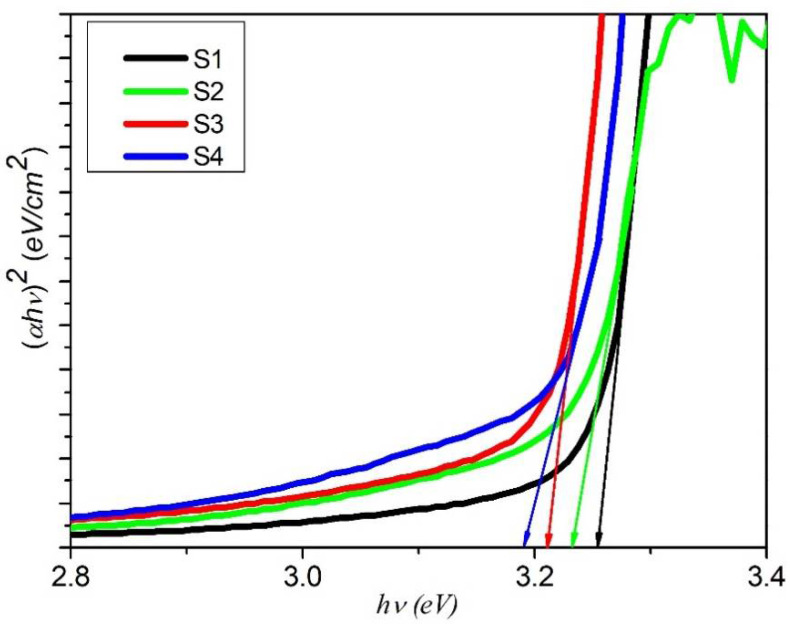
Band gap energies of Cu- and Sm-doped ZnO nanorod arrays.

**Figure 7:**
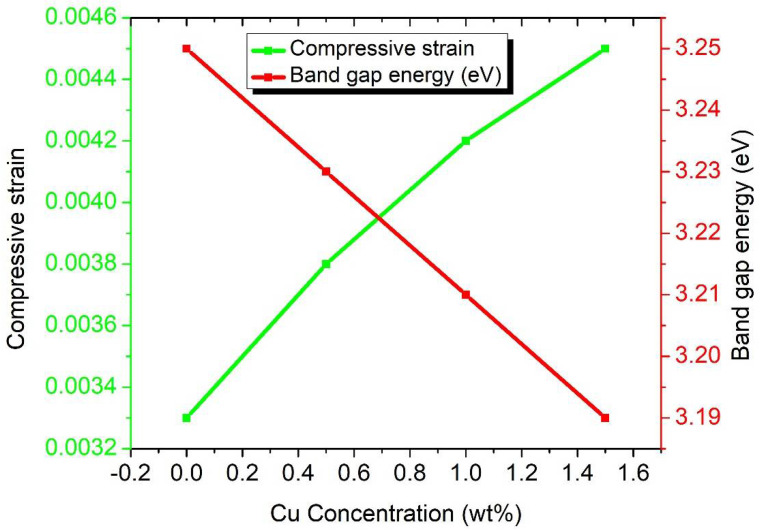
Compressive strain and band gap energy vs. Cu concentration of ZnO nanorod arrays.

**Figure 8:**
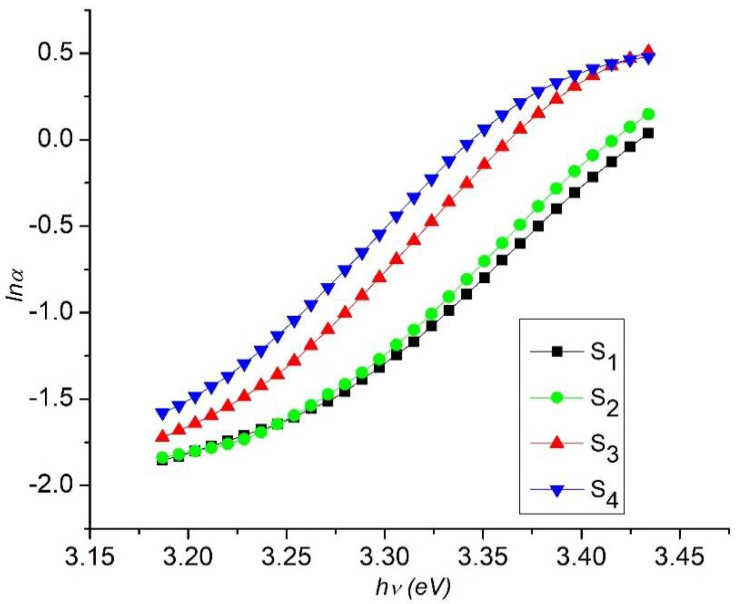
Plot of ln ln (α) vs Photon energy (hν) of Cu- and Sm-doped ZnO nanorod arrays.

Figure 5 illustrates the optical absorbance spectra of Cu- and Sm-doped ZnO nanorod arrays films after the sensitization of N719 dye. It is observed that absorption in the visible wavelength range of the light spectrum increases with the increase in the Cu concentration and Sm precursor. As the concentration of dopants increases, morphology of uniform size nanorod arrays is obtained with high porosity and large surface area (sample S_4_). The enhancement in absorption is attributed to the transfer of charge between the conduction or valence band of ZnO and the 4f level of Sm^3+^ ions. The absorption peak of 0.5 wt% of Cu- and 1 wt% of Sm-doped ZnO nanorod arrays is found about 521 nm in the visible region of the light spectrum. At the same time, for other doping levels 1.0 wt% and 1.5 wt% of Cu and 1 wt% of Sm, the intensity of absorption peaks was increased. As the concentration of dopants increases, an enhancement in the absorption is observed, and the absorption band moves towards the green emission. Fons, Wahl and their coworkers have explained that as Cu belongs to IB group elements and can act as an accepter in ZnO [42,43,44]. They think that Cu-related defects are of great importance and are the main cause for the green emission instead of the intrinsic defects such as oxygen vacancies. In addition, this increment in absorption is also associated with the formation of localized states in the ZnO band gap and confirmed that ZnO nanorod arrays have been modified with Cu/Sm dopants. The band gap energies of the as deposited nanorod arrays were calculated before anchoring N719 dye using Tacu plot relation as shown in Equation (5) [37]:

(5) $${\left(\alpha hv\right)}^{2}=A\left(hv-E_{g}\right)$$

where *α*, *hν*, *A* and *E*_g_ are the absorption co-efficient, photon energy, constant and band gap energy, respectively. Figure 6 delineates the plot between the photon energy hν and the absorption coefficient, (*αhv*)^2^, and the calculated values are given in Table 2. The estimated values of band gap energy are observed to decrease from 3.25 eV to 3.19 eV as we increase the dopant concentration from 0.0 wt% to 1.5 wt%. The reduction in band gap energy of semiconductors is worth noticing by doping with transition metals or rare earths. By doping transition/rare earth metals, new energy levels formed in the band gap, which decreases the band gap *E*_g_ of ZnO [45]. The formation of new energy levels near the conduction band is due to the donor impurities and near the valence band is due to the acceptor impurities. When the amount of dopant elements is increased, then the density of their states is also increased and forms a continuum of states just like in the bands, as a result *E*_g_ is decreased. Band gap energy and compressive strain vs. Cu concentration of ZnO nanorod arrays for different samples are presented in Figure 7. The absorption coefficient of ZnO nanorod arrays shows a tail for sub band gap photon energies. This tail is called Urbach tail and is closely related to the disorder in the film network. The Urbach tail is expressed as [37]:

(6) $$\alpha =\alpha_{o}exp\left(\frac{h\nu }{E_{u}}\right)$$

where $\alpha_{o}$ is a constant and *E*_u_ is Urbach energy which characterizes the slope of the exponential edge. The above equation delineates the optical transition between occupied state in the valence band tail to unoccupied state of the conduction band edge. The values of *E*_u_ can be calculated from the inverse of the slope of ln*α* versus (*hν*). Urbach energy values change inversely with optical band gap, i.e., with the increase in Cu concentration and Sm precursor the Urbach tail increases from 85 to 100 meV as shown in Figure 8.

### 3.4. I-V Measurements of Cu-Doped ZnO-Sm Films

**Figure 9:**
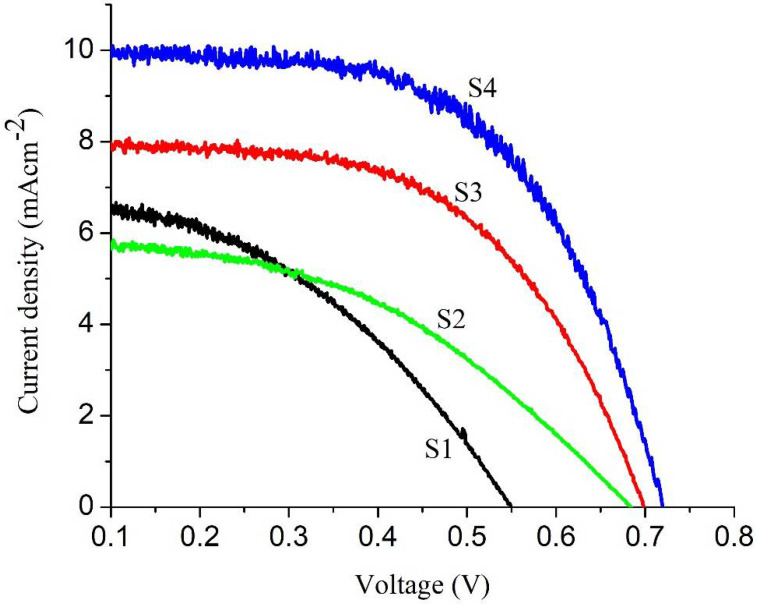
I-V characteristics of Cu- and Sm-doped ZnO nanorod arrays.

Figure 9 depicts the current-voltage response of DSSCs fabricated with different samples (S_1_ to S_4_) of Cu- and Sm-doped ZnO nanorod arrays photoanodes. Completed cells were illuminated by 1 sun AM 1.5 G, and all the currents and voltages (*I*_sc_, *V*_oc_, *I*_max_, *V*_max_) are measured and listed in Table 3. The efficiency (*η*) and fill factor (FF) are calculated from Equations (7) and (8) [46]:

(7) $$\eta \;\left(\%\right)=\frac{P_{max}}{I_{rad}\times A}\times 100$$

(8) $$FF\;\left(\%\right)=\frac{V_{max}\times I_{max}}{V_{oc}\times I_{sc}}\times 100$$

where *P*_max_, *I*_rad_ and *A* are the maximum power output, the input light and the working area of the cell, respectively. The measurements show that the cell fabricated with 0.5 wt% of Cu- and Sm-doped ZnO nanorod arrays have superior open circuit voltage (*V*_oc_) = 0.681 *V* and efficiency (*η*) = 2.47% than the cell fabricated with 0.0 wt% of Cu- and Sm-doped ZnO nanorod arrays, (*V*_oc_) = 0.550 V and *η* = 1.74%. The significant enhancement in *V*_oc_ and *η* is because of the presence of Cu/Sm, which impeded the recombination rate of electrons and increased the transport of electrons. There is an improvement of 27% in efficiency. It has been reported that some rare earth ion modifications can passivate the surface states of the ZnO electrode. For instance, modification with Sm, Gd and Nd ions particularly boosted the open-circuit photovoltage and fill factor of ZnO-based solar cells and decreased short-circuit current [47,48,49], which is in accordance with our results. The further improvement in *J*_sc_ = 26% and *η* = 55%, has been observed in DSSCs fabricated with 1.0 wt% of Cu and 1 wt% of Sm-doped ZnO nanorod arrays. The maximum photocurrent density and efficiency has been recorded for the DSSC fabricated with 1.5 wt% of Cu- and 1 wt% of Sm-doped ZnO nanorod arrays (i.e., *J*_sc_ = 42% and *η* = 60%, respectively), as indicated in Table 3. The enhancement in cell parameters can be explained as follows: when ZnO is doped with rare earth Sm^3+^ ions, an up/down conversion process takes place, in which ultraviolet and near infrared radiations are shifted to the visible light region. In this way, two or more low energy photons can be absorbed by ZnO, which in turn results in the form of emission of high energy photons present in the core absorption region of the N719 dye [50]. As reported in the previous literature, N719 dye has strong absorption at about 550 nm [51]. This broadening of light absorption region is the key factor to boost power conversion efficiency of DSSCs. Moreover, doping of rare earth ions in ZnO also acts as a blocking layer that hinders charge recombination among iodide/triiodide $I^{-}/I_{3}^{-}$ electrolyte and photoelectrode and enhance the injection of excited electrons [52]. Another way to increase the performance of DSSCs based on 0.5 wt%, 1.0 wt% and 1.5 wt% of Cu- and Sm-doped ZnO nanorod arrays by decreasing the backward transfer of electrons. Doping of Cu in ZnO also creates a blocking layer which prevents the backward transfer of electron, and the electron easily moves towards the molecules of oxidized dye or liquid electrolyte [30]. In general, doping of ZnO with coupled metals Cu and Sm modifies the flat-band potential auspiciously to enable more efficient injection of charge carriers at the interface of ZnO and dye, thereby enhancing the overall performance of DSSCs [29,30,31,32,33].

## 4. Conclusions

The present study demonstrates the synthesis of Cu-and Sm doped ZnO nanorod arrays with a fixed concentration of Sm (1 wt%) and different concentrations of Cu (0.0, 0.5, 1.0 and 1.5 wt%). The effect of Cu concentration and Sm precursor on structural, optical, morphological and photovoltaic properties have been studied. XRD pattern showed that the nanorod arrays were crystalline in nature and have hexagonal wurtzite structure. In UV-Vis analysis, the absorbance increases with the increase in Cu/Sm content and band gap decreases from 3.25 eV to 3.19 eV. I-V characteristics of DSSCs revealed that the cell fabricated with 1.5 wt% of Cu-and 1 wt% of Sm doped ZnO nanorod array photoanodes have maximum efficiency of 4.14%, which is about 60% higher when compared to their other counterparts. The enhancement in the efficiency of dye-sensitized solar cells is attributed to the doping of ZnO with coupled metals Cu and Sm, which modifies the flat-band potential auspiciously to enable more efficient injection of charge carriers at the interface of ZnO and dye, thereby enhancing the overall performance of DSSCs. This work strongly supports the coupled use of transition and rare earth metals for further development of photovoltaic device applications.

## Figures and Tables

**Table 1:** Parameters specifying fabrication of Cu- and Sm-doped ZnO nanorod arrays.

Samples	Zn_1−*x*−*y*_Sm*_x_*Cu*_y_*O	Content of Sm (wt%)	Content of Cu (wt%)
S_1_	Zn_1−*x*−*y*_Sm*_x_*Cu*_y_*O	1	0.0
S_2_	Zn_1−*x*−*y*_Sm*_x_*Cu*_y_*O	1	0.5
S_3_	Zn_1−*x*−*y*_Sm*_x_*Cu*_y_*O	1	1.0
S_4_	Zn_1−*x*−*y*_Sm*_x_*Cu*_y_*O	1	1.5

**Table 2:** Calculated parameters from XRD graph of ZnO (002) peak and band gap energy values.

Samples	FWHM (*β*)	Crystallite Size (*D*) (nm)	Compressive Strain (ε)	d-Spacing (*d*) (nm)	Dislocation Density (δ) (nm)^−2^	Band Gap (*E*_g_) (eV)
S_1_	0.2385	36.48	0.0033	0.256	0.0007	3.25
S_2_	0.2714	32.07	0.0038	0.257	0.0010	3.23
S_3_	0.3001	28.99	0.0042	0.258	0.0012	3.21
S_4_	0.3261	26.68	0.0045	0.260	0.0014	3.19

**Table 3:** I-V measurement of Cu- and Sm-doped ZnO nanorod arrays DSSCs.

Cell Parameters	Samples			
	S_1_	S_2_	S_3_	S_4_
*J*_sc_ (mA/cm^2^)	6.32 ± 0.046	5.84 ± 0.017	7.87 ± 0.021	9.99 ± 0.011
*V*_oc_ (V)	0.550 ± 0.002	0.681 ± 0.005	0.692 ± 0.004	0.725 ± 0.003
FF	0.43 ± 0.003	0.51 ± 0.006	0.67 ± 0.003	0.63 ± 0.004
*η* (%)	1.74 ± 0.001	2.47 ± 0.012	3.30 ± 0.002	4.14 ± 0.001

## Data Availability

The raw data used for this proposed work have been cited in the manuscript. Moreover, the derived data supporting the findings of this study have been graphically depicted and are available from the corresponding author on request.
